# Isolation of Rhp-PSP, a member of YER057c/YjgF/UK114 protein family with antiviral properties, from the photosynthetic bacterium *Rhodopseudomonas palustris* strain JSC-3b

**DOI:** 10.1038/srep16121

**Published:** 2015-11-04

**Authors:** Pin Su, Tuizi Feng, Xuguo Zhou, Songbai Zhang, Yu Zhang, Ju’e Cheng, Yuanhua Luo, Jing Peng, Zhuo Zhang, Xiangyang Lu, Deyong Zhang, Yong Liu

**Affiliations:** 1 Key Laboratory for the Integrated Management of Pest and Disease on Horticultural Crops in Hunan Province, Hunan Plant Protection Institute, Hunan Academy of Agricultural Sciences, Changsha 410125, China; 2 Department of Entomology, University of Kentucky, Lexington, KY, 40546, USA; 3 College of Bioscience and Biotechnology, Hunan Agricultural University, Changsha 410128, China

## Abstract

*Rhodopseudomonas palustris* strain JSC-3b isolated from a water canal adjacent to a vegetable field produces a protein that was purified by bioactivity-guided fractionation based on ammonium sulfate precipitation, ion-exchange absorption and size exclusion. The protein was further identified as an endoribonuclease L-PSP (Liver-Perchloric acid-soluble protein) by shotgun mass spectrometry analysis and gene identification, and it is member of YER057c/YjgF/UK114 protein family. Herein, this protein is designated Rhp-PSP. Rhp-PSP exhibited significant inhibitory activities against tobacco mosaic virus (TMV) *in vivo* and *in vitro*. To our knowledge, this represents the first report on the antiviral activity of a protein of the YER057c/YjgF/UK114 family and also the first antiviral protein isolated from *R. palustris*. Our research provides insight into the potential of photosynthetic bacterial resources in biological control of plant virus diseases and sustainable agriculture.

Tobacco mosaic virus (TMV) is one of the most destructive plant viruses and can infect nine plant families and at least 125 individual species worldwide. TMV causes severe yield loses in most agricultural areas^1^. Physiochemical methods are widely used to control the disease, but food safety and environmental concerns require alternatives or supplemental methods to overcome the negative effects of chemical pesticides, such as resistance, pesticide-residues and phytotoxicity. One approach to meeting these requirements is the selection of antiviral metabolites from nature for direct utilization in virus control or as lead compounds for the development of new bio-resourced pesticides. Extensive work has been directed towards the isolation and identification of candidate compounds with antiviral properties from beneficial microorganisms, and the resultant bio-control agents exhibit significant and efficacious control of plant viruses in the field. Thus far, these beneficial microorganisms are limited to a relatively small group that primarily consists of *Bacillus spp*^2^,^3^, *Pseudomomas spp*^4^, *Actinomyces spp*^5^ and some fungal strains represented by *Trichoderma harzianum*^6^.

Photosynthetic bacteria are a phylogenentically diverse group that is unified by the capacity to perform bacteriochlorophyll (Bchl)-based photosynthesis^7^. These bacteria are also well-known due to their wide distribution in bodies of water and their versatile nitrogen fixation, carbon sequestration, hydrogen production and desulfurization abilities^8^,^9^. The applications of these bacteria range from bacterial fertilization, sewage treatment, and hydrogen production to chemical pesticide degradation^10^,^11^,^12^ and even medical uses^13^. Despite the multifunctional roles of these bacteria in the agricultural and environmental protection fields, their abilities to control viruses remain largely unstudied and underexploited. Our previous laboratory and field tests revealed that the photosynthetic bacteria *Rhodopseudomonas palustris* strain JSC-3b^14^, which had previously been characterized as a strain with the potential to biodegrade pyrethroid residues in the field, exhibits a strong suppression of TMV in tobacco. Specifically, when the supernatant of its enrichment liquid was applied to tobacco leaves, the incidence of TMV remained significantly lower than that of mock controls in field tests. Thus, we hypothesise that an active metabolite produced by JSC-3b played a role in TMV suppression. In the present study, the antiviral compound was isolated and identified as an endoribonuclease L-PSP protein that we designated Rhp-PSP. Based on the genomic sequence of *R. palustris* JSC-3b, the Rhp-PSP protein contains 152 amino acids and was assigned to the YER057c/YjgF/UK114 protein family.

## Results

### Bioactivity-guided isolation and purification

**Figure 1:**
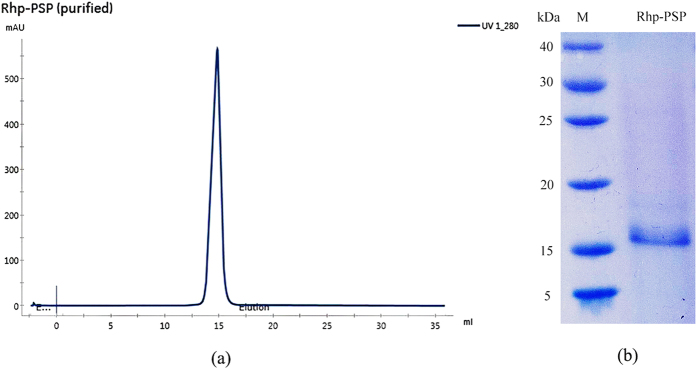
Purification of Rhp-PSP from *R. palustris* JSC-3b. (**a**) The active peak from size-exclusion chromatography using a HiPrep 16/60 Sephacryl S100 HR column was collected and loaded onto Superdex 200 Increase 10/30 GL column to obtain a single peak (Elution volume = 14.87 mL). (**b**) Tricine SDS-PAGE analysis of the purified Rhp-PSP revealed a single band with Coomassie brilliant blue R-250 staining at a size of slightly greater than 15 kDa. Lane: M, protein molecular mass marker.

To identify the protein that exhibited the antiviral activity, protein samples were tested for their abilities to inactivate TMV particles following premixing and incubation with a TMV particle solution *in vitro*. The inactivated TMV particles were identified by their inability or attenuated ability to infect tobacco leaves. Thus, the tobacco leaves presented fewer or no lesions after inoculation with the mixtures contained proteins that exerted inactivation effects on TMV. The crude protein samples (i.e., protein fractions) obtained from each isolation procedure were subjected to the half-leaf method to observe their inactivation effects on TMV as described in the bioactivity-guided fractionation section of the material and methods. Guided by the bioactivity results, we found that the precipitated protein began to exhibit inactivation effects when the relative saturation of ammonium sulfate reached 60%, and the effect was enhanced with increases in the relative saturation until the saturation reach 70%. Thus, the target protein began to precipitate at a relative ammonium sulfate saturation of 60%, and precipitation did not proceed after the relative saturation exceeded 70%. Thus, the untargeted proteins that precipitated at 55% were discarded, and this process greatly simplified the subsequent purification procedure. The desalted protein fraction with bioactivity was subjected to anion-exchange chromatography using a HiTrap Q FF column for linear gradient elution, and the active peak was collected and then subjected to size-exclusion chromatography using a HiPrep 16/60 Sephacryl S100 HR column. The active peak from the size-exclusion chromatography was loaded onto the Superdex 200 Increase 10/30 GL column (with higher efficiency than HiPrep 16/60 Sephacryl S-100 HR column for separation of single protein from mixed sample) to ensure its purity (Fig. 1a) and exhibited a single band on Tricine SDS-PAGE with a relative apparent molecular mass of 16 kDa (Fig. 1b). After ultrafiltraion concentration and vaccum freeze-drying of pooled fractions with target protein, total 70.5 mg of pure compound was obtained from 5 L of *R. palustris* JSC-3b cultured medium.

**Figure 2:**
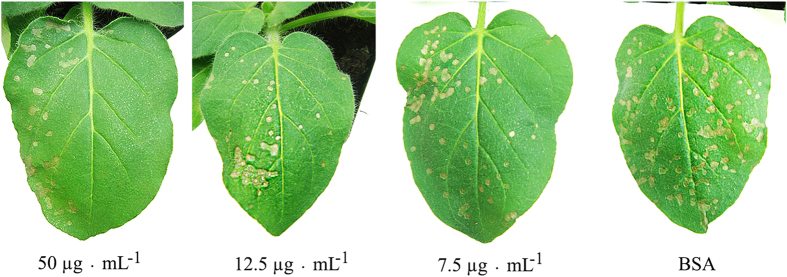
The inactivation effect of Rhp-PSP on TMV. The purified Rhp-PSP was diluted to concentrations of 50, 12.5 and 7.5 μgmL^−1^ to test the inactivation effects on TMV. BSA at a concentration of 50 μgmL^−1^ was used as the negative control. The right hand sides of the leaves were treated with a mixture of protein diluents and TMV, and the left hand sides were treated with buffer control. Photographs were taken three days after treatment.

The purified protein was diluted to concentrations of 500, 250, 125, and 75 μgmL^−1^ as described in the bioactivity-guided fractionation section to test its inactivation effect on TMV. The observations from the half-leaf method revealed that the *Nicotiana glutinosa* leaves inoculated with the protein-treated TMV exhibited no apparent lesions. To observe the concentration-dependent inactivation effects of the protein, the protein was subsequently diluted to lower concentrations of 50, 12.5, and 7.5 μgmL^−1^ that were then used in the inactivation effect test. The inactivation effect still achieved a level of 82% even though the concentration was lowered to 7.5 μgmL^−1^, according to the results (Fig. 2).

### Mass spectrometry analysis and cloning of Rhp-PSP gene

**Figure 3:**
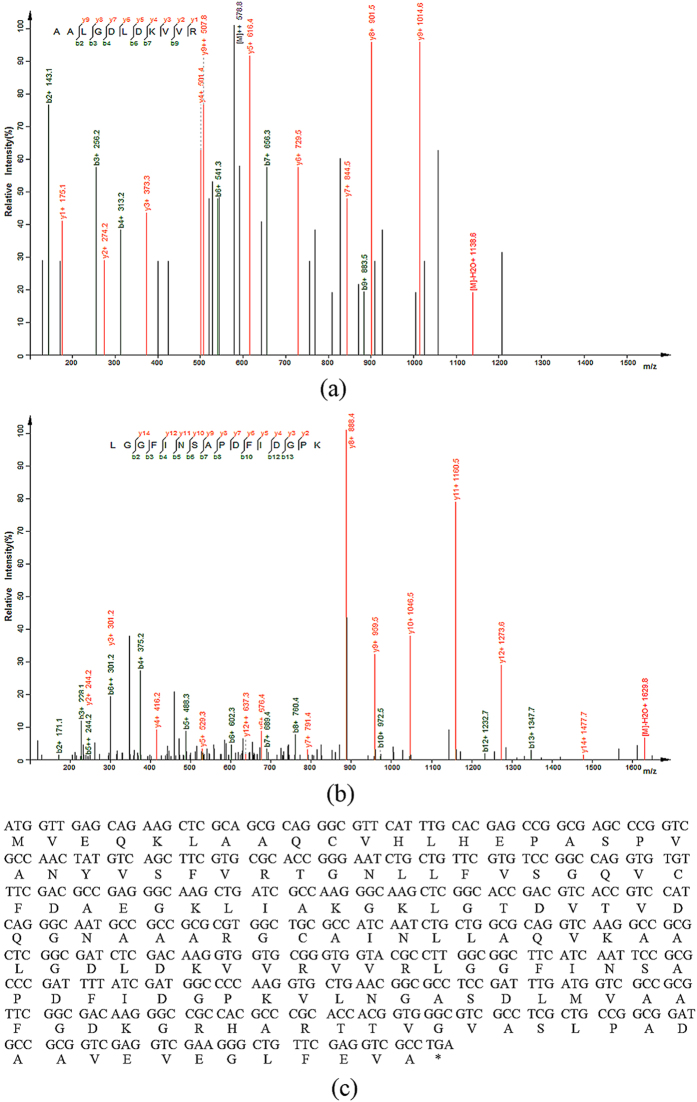
The liquid chromatography (LC)-MS/MS analysis and the deduced sequence of Rhp-PSP. (**a,b**) Two peptides, AALGDLDKVVR and LGGFINSAPDFIDGPK, were identified with 95% confidence. (**c**) The nucleotide sequence of the PCR products of Rhp-PSP was identical to the sequence from the genome of *R. palustris* JSC-3b. The stop codon TGA is shown as an asterisk in the deduced amino acid sequence.

The protein sample was excised from the Tricine SDS-PAGE gel for liquid chromatography (LC)-MS/MS analysis of the in-gel-digested protein to determine the amino acid sequence of Rhp-PSP. The MS/MS data were analysed as described in the shotgun mass spectrometry analysis section of the materials and methods. Based on the ProteinPilot results, we obtained the best-matching protein, which was endoribonuclease L-PSP (UniProt No. Q07L61), from the *R. palustris* strain BisA53. The peptides coverage of the match was 76% with 39 different reliable peptides. Here, we present two of those peptides, i.e., AALGDLDKVVR (Fig. 3a) and LGGFINSAPDFIDGPK (Fig. 3b). Using the genomic sequence of JSC-3b, we found the endoribonuclease L-PSP gene, which had a full-length of 459 bp and encoded a protein of 152 amino acids (Fig. 3c). The gene was then cloned from the JSC-3b strain genomic DNA and verified by sequencing. The results revealed an 85.26% similarity with the gene from strain BisA53 based on alignment (DNAMAN, Version 8.0). Therefore we designated new protein isolated from *R. palustris* strain JSC-3b as Rhp-PSP.

### Protective and curative effects of Rhp-PSP against TMV

**Figure 4:**
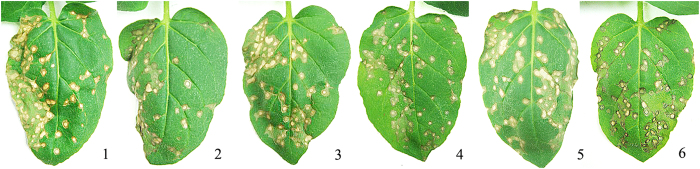
The protective and curative effects of Rhp-PSP against TMV on *N. glutinosa*. Photo 1, 2 were protective and curative effects of Rhp-PSP; photo 3, 4 were of Ningnanmycin; photo 5, 6 were of BSA. All chemicals were applied at the concentration of 100 μgmL^−1^. The right hand sides of the leaves were treated with chemicals, and the left hand sides were treated with buffer control. Photographs were taken seven days after TMV inoculation.

Based on the bioactivity-guided isolation and purification, we knew that TMV was inactivated by Rhp-PSP *in vitro*. To investigate whether TMV was inhibited by Rhp-PSP *in vivo*, tobacco leaves were treated with Rhp-PSP diluents without premixing and incubated with TMV particles. As described in the anti-TMV assays of Rhp-PSP section, the tobacco leaves were treated with the protein before TMV inoculation to observe whether Rhp-PSP protected the tobacco from TMV infection, and the leaves were treated after TMV inoculation to observe whether Rhp-PSP halted disease development in the infected tobacco leaves. The leaves from both treatments would exhibit no or fewer lesions if the Rhp-PSP exhibited protective or curative effects against TMV. The commercialized antiviral chemical Ningnanmycin was employed for comparison with the effects of Rhp-PSP. As shown in Table 1, both Rhp-PSP and Ningnanmycin exhibited significant and concentration-dependent protective and curative effects against TMV. At the same concentrations, Rhp-PSP exerted significantly better curative and protective effects than Ningnanmycin. Specifically, at the concentration of 100 μgmL^−1^, Rhp-PSP elicited a protective effect of 76.5% and a curative effect of 78.3%, whereas these values for Ningnanmycin were 61.0% and 54.5% (Fig. 4). At both concentrations, the mock control BSA exhibited no obvious effects in this experiment.

## Discussion

Based on sequence similarity, the antiviral protein Rhp-PSP produced by *R. palustris* strain JSC-3b was identified as a putative endoribonuclease L-PSP and a member of the highly conserved YER057c/YjgF/UK114 protein family. Homologues of this family are widely distributed in eubacteria, archaea and eukaryotes. Although the sequences and structures of these homologues exhibit high levels of similarity, the functions vary widely across different species^15^. The elucidations of the crystal structures of homologues from *Escherichia coli*, *Bacillus subtilis*, *Saccharomyces cerevisiae*, *Pseudomonas syringae*, rats, goats, humans and other species have revealed that the proteins of this family exhibit highly similar homotrimeric structures^16^ and that the conserved domains are also similar in structure to the bacterial chorismate mutase despite the absence of sequence similarity or a functional connection^17^. Rat liver perchloric acid-soluble protein (RL-PSP) was the first member of this family to be isolated and was characterized as an endoribonuclease^18^. RL-PSP inhibits protein translation in reticulocyte cell-free systems by directly affecting mRNA template activity and induces the disaggregation of reticulocyte polysomes into 80 S ribosomes^19^. The homologue of RL-PSP hp14.5 was subsequently found in human monocytes and was also characterized as a translational inhibitor that is up-regulated during cellular differentiation^20^. The homologue UK114 from goat liver has been reported to exhibit tumour antigen activity^21^, and a bovine homologue elicits calpain activation^22^. In addition to the mammalian homologues, a series of proteins from this family with diverse functions have been identified in lower eukaryotes and bacteria, such as the purine regulator YabJ from *B. subtilis* YabJ^23^, YIL051c and YER057c from *S. cerevisiae,* which are involved in isoleucine biosynthesis and the maintenance of intact mitochondria^24^, and the YjgF family of proteins in plants, which are involved in photosynthesis and chromoplastogenesis (CHRD)^25^. Other functions, such as fatty acid-binding^26^ and the repression of cell proliferation have also been observed^27^. Based on the discovery and functional description of these homologues, the proteins from this family are considered to be multifunctional, but no common biological activity has yet been attributed to them^28^,^29^. The determined quaternary structures of the YER057c/YjgF/UK114 family proteins suggest that a group of ligand binding sites exist in the clefts between the monomeric subunits^30^ and could potentially enable the proteins to bind various substrates and ligands, such as benzoate^31^, acetate^23^, 2-ketobutyrate^16^, free fatty acids^26^ and D-glucose^32^. These binding sites are highly conserved in all members of the YER057c/YjgF/UK114 family, and their binding activities could enable the proteins to modulate their functions in the presence of different metabolites^15^,^33^. Combined with the high levels of similarity in sequence and structure, the strong conservation of the binding sites enables the presumption that these proteins use the same biochemical mechanism to manifest their biological functions^33^. This presumption is supported by experimental results that have indicated that diverse members of the YER057c/YjgF/UK114 family proteins, i.e., the human homologue UK114 and the *Salmonella enterica* homologue YjgF, inhibit anthranilate phosphoribosyltransferase (TrpD)-dependent phosphoribosylamine (PRA) synthesis *in vitro*^34^.

**Figure 5:**
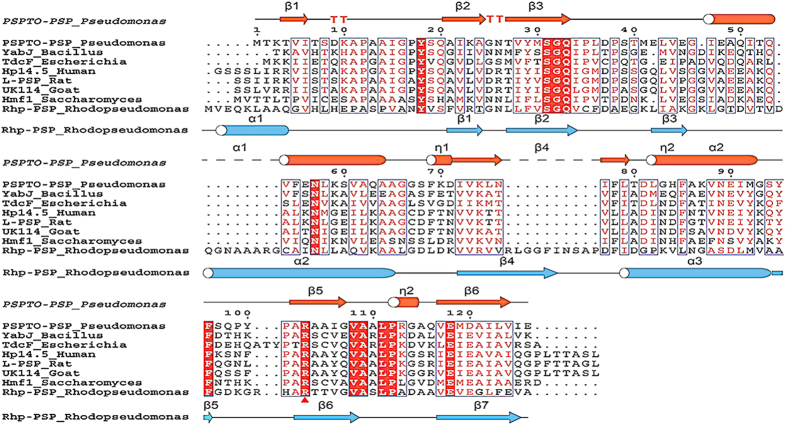
Multiple sequence alignment of the YER057c/YjgF/UK114 family. The last sequence shown in the picture is the deduced sequence of Rhp-PSP. The twelve invariant residues are highlighted in red, and the Arg^129^ of Rhp-PSP is indicated by the red triangle under the Rhp-PSP sequence. The sequences of the homologues and their crystal structures were obtained from UniProt and protein data bank RCSB PDB. This alignment and presentation was performed using ClustalX2 and ESPpript 3.0, and the secondary structure of Rhp-PSP was predicted in JPred 4.

In our present study, we initially isolated the putative endoribonuclease L-PSP from *R. palustris* and revealed its antiviral properties both *in vivo* and *in vitro*. However, due to the lack of direct evidence regarding the mechanism of the antiviral properties of this protein and the fact that most of its homologues are hypothetical proteins with unknown functions^28^, it seemed difficult to deduce the possible mechanism and support our results based on known resources related to this protein. However, its homologue RL-PSP has been reported to inhibit the protein translation of the tobacco mosaic virus mRNA in a rabbit reticulocyte lysate system, and it was subsequently proven the inhibition was driven by endoribonucleolytic activity, which cleaves the mRNA in a different manner than RNase A^19^. The multiple sequence alignment (Fig. 5) of the deduced sequence of Rhp-PSP with its homologues, i.e., PSPTO-PSP from *P. syringe*, YabJ from *B. subtilis*, TdcF from *E. coli*, Hp14.5 from humans, L-PSP from rats, UK114 from goats, and Hmf1 from *S. serevisiae* revealed that twelve residues of the YER057c/YjgF/UK114 family are invariantly conserved. Among these invariant residues, the conserved Arg^103^ of PSPTO-PSP and Arg^107^ of hp14.5 (which align with the Arg^129^ of Rhp-PSP) have been reported to be the crucial amino acids for different ligands and suitable binding sites for ribonucleotides^32^.

As typified by ribonucleases, RNA-degrading enzymes catalyse the degradation of RNA into smaller components and can result in the degradation of viral RNA in certain plants. This process may ultimately block viral replication and symptoms causes by viruses^2^,^35^,^36^. Given the above information and the speculation that Rhp-PSP also possesses the properties of an endoribonuclease L-PSP, we hypothesised that Rhp-PSP may exert its anti-TMV activity through the degradation of the RNA of this positive-sense single-stranded RNA virus. However, the study related to the endoribonucleolytic activities of the YER057c/YjgF/UK114 family is minimal and limited to the homologues from humans, rats, goats, and the newly reported EhL-PSP from *Entamoeba histolytica*^37^. The *R. palustris*-produced endoribonuclease L-PSP has not yet been characterized. Therefore, to understand the molecular mechanism underlying the antiviral activity of Rhp-PSP, our next studies will focus on providing the *de facto* data demonstrating the endoribonuclease activity of this protein and investigating how such activity functions in TMV suppression both *in vivo* and *in vitro*.

## Conclusions

We have reported the purification and gene cloning of an antiviral protein named Rhp-PSP that is produced by *R. palustris* strain JSC-3b. This protein was identified as a putative endoribonuclease L-PSP of the YER057c/YjgF/UK114 family. Rhp-PSP exhibited strong inactivation effects *in vitro* and also exhibited protective and curative effects against TMV in *N. glutinosa*. This study is the first to reveal a TMV-suppressing effect of a member of the YER057c/YjgF/UK114 family, and thus this study provides an initial indication of the potential application of this protein in bio-control and sustainable agriculture.

## Materials and Methods

### Microorganisms, plants and culture conditions

*R. palustris* JSC-3b (Genbank accession number: AYSU00000000) isolated from a water canal adjacent to a vegetable field at 28°11’49” N, 112°58’42” E was cultured in medium containing the following (gL^−1^): (NH_4_)_2_SO_4_ 0.1, MgSO_4_ 0.02, Na_2_CO_3_ 0.5, K_2_HPO_4_ 0.05, NaCl 0.02, and yeast extract 0.15 (pH = 6.5–7.0). The seed cultures were prepared in a 250-ml Erlenmeyer flask containing 250 ml of medium that was cultivated at 30 °C and 6,500 lux in a light incubator (PRX-450D, China) for 5 days, and the flask was manually shaken 3–5 times per day. Five 1-L Erlenmeyer flasks were used for the-large scale production of the protein in the same medium under the same temperature and light intensity conditions but without an initial pH control. The inoculation volume was 5% (v/v) for each flask, and the incubation time was 7 days.

The TMV U1 strain was propagated and purified from its systemic host *Nicotiana tabacum* cv. Huangmiaoyu using the following method. One hundred gram of infected leaves were ground for 2 minutes in the presence of 200 ml of buffer A (pH = 7.5, 0.5 M PB buffer containing 0.01 M Na_2_EDTA and 0.1% mercaptoethanol) in a pulp refiner, and the liquid was then filtered with double-layer gauze. The filtrate was centrifuged (6,000 rpm, 20 minutes), and the supernatant was collected. Triton X-100, PEG 6000 and NaCl were slowly added to concentrations of 2.5%, 4% and 0.1 M, respectively, during manual agitation. The mixture was stirred for 4 hours at a temperature of 4 °C using a magnetic stirrer and subsequently centrifuged (11,000 rpm, 15 minutes). The precipitate was re-suspended with buffer B (pH = 7.5, 0.5 M PB buffer containing 0.01 M MgCl_2_ and 0.5 M urea) and then centrifuged (6,000 rpm, 15 minutes). The supernatant was collected, the precipitate was processed with the same method three times, and the supernatants from each step were mixed. The mixed supernatant was centrifuged (32,000 rpm, 107 minutes), and the precipitate was re-suspended in PB buffer (pH = 7.5, 0.5 M). The suspension was centrifuged (8,000 rpm, 15 minutes), and the supernatant was collected for sucrose density gradient centrifugation. Step gradients were prepared by layering 5 ml of 10%, 5 ml of 20%, 6 ml of 30%, 6 ml of 40% and 2 ml of 50% sucrose dissolved in buffer C (pH = 7.5, 0.5 M PB buffer containing 0.01 M MgCl_2_). The supernatant (2 ml) was layered on top of the gradient and then centrifuged (32,000 rpm, 107 minutes). The TMV particle-containing fraction was pooled and diluted with the same volume of buffer C. The diluted fraction was again centrifuged (45,000 rpm, 90 minutes), and each pellet was re-suspended in 1 ml of buffer C. This suspension was designated as the purified TMV particle suspension and preserved at a temperature of −20 °C for bioactivity-guided fractionation. The concentration of the purified TMV suspension was determined with a Nano DROP 2000 C Spectrophotometer (Thermo Scientific, Thermo Fisher Scientific Inc., USA).

The TMV necrotic host *N. glutinosa* was used in the bioactivity assay. Tobacco seedlings were grown in flowerpots (d = 20 cm) with a photoperiod of 16 h of light at 25 °C and 8 hours of dark at 18 °C, light intensity of 5,000 lux and approximately 50% relativity humidity. The seedlings with 6–8 leaves were selected for the treatments. All seedlings that were used in the experiments were healthy and had not been infected by any pathogens.

### Bioactivity-guided fractionation

To determine the inactivation effects of the protein extracts on TMV, purified TMV particles and pure compounds or protein fractions were subjected to the half-leaf method on *N. glutinosa* as described by Kunihiro Kasamo and Toru Shimomura (1978)^38^ with minor modifications. The pure compounds or fractions were dissolved in phosphate buffer (PB, pH = 7, 0.2 M) to a concentration of 1 mgmL^−1^ and twofold serially diluted to final concentrations of 500, 250, 125, and 75 μgmL^−1^. The same concentrations gradient of bovine serum albumin (BSA) was prepared for the negative control treatments. The purified TMV particles was diluted to 1 μgmL^−1^ with the same PB solution mentioned above. The same volumes (50 μl) of the TMV and protein solutions were mixed. After 10 minutes, 20 μL of the mixture was rubbed onto the right hand sides of the leaves of the tobacco, and the left hand sides of the leaves were inoculated with the mixture of PB and TMV particles solution as a blank control. The fourth and fifth leaves on each plant were selected and sprinkled with carborundum for inoculation. The local lesion numbers were recorded 3 days after inoculation.

The inactivation effects of the protein were calculated according to the following formula: inactivation effect (%) = [(C − T)/C] × 100%. C indicates the average local lesion number on the control, and T indicates the average local lesion number on the protein extract-treated leaves.

### Protein extraction and purification

*R. palustris* strain JSC-3b was cultured in 5 L of medium to produce the target protein as described in the microorganisms, plants and culture conditions section. The supernatant was collected after centrifugation (8,000 rpm, 15 minutes) and was then pump-filtered through a 0.22-μm micro-membrane (Merck Millipore Ltd., Tullagreen, Carrigtwohill Co. Cork, IRELAND) to remove the residual bacteria. Solid ammonium sulfate was added to the supernatant to achieve 55% (wt/vol) relative saturation at 4 °C overnight. The protein that precipitated following the 55% relative saturation with ammonium sulfate was separated by centrifugation (11,000 rpm, 15 minutes) and discarded. Solid ammonium sulfate was subsequently added to the remaining supernatant to achieve 75% (wt/vol) relative saturation at 4 °C overnight. The precipitate was then harvested by centrifugation (11,000 rpm, 15 minutes) and re-dissolved in 50 ml of buffer D (PB, pH = 6.7, 0.02 M). After filtering the crude protein with a 0.22-μm filter, the filtrate was loaded onto a HiTrap desalting column with elution buffer D. The desalted protein solution was loaded onto an anion-exchange chromatography HiTrap Q FF column that had been pre-equilibrated with elution buffer (Tris-HCl, pH = 8.0, 0.02 M). The bound proteins were eluted with a linearly increasing gradient of NaCl in elution buffer at a flow rate of 1 ml/min. All fractions were collected and desalted in a column for the TMV inactivation tests. The active fraction after desalting was loaded onto a size-exclusion chromatography HiPrep 16/60 Sephacryl S100 HR column with buffer D as the elution buffer. Fraction collectors collected each peak automatically for the TMV inactivation tests. The active fraction was then subjected to an additional size-exclusion chromatography with the column Superdex 200 Increase 10/30 GL to ensure its purity, and the molecular mass was determined via Tricine SDS-PAGE. The purified protein fractions from multiple parallel purification procedures were pooled and concentrated by centrifugation (6,000 rpm, 45 mins) using Millipore AMICON ULTRA 15 ML 3 K NMWL 8PK (Merck Millipore Ltd., Tullagreen, Carrigtwohill Co. Cork, IRELAND), and quantified by Bradford method before vaccum freeze-dried for storation.

The chromatographic fractionations were performed on a NGC^TM^ Scout 10 Plus Chromatography system (Bio-Rad Laboratories, Inc., USA) equipped with columns from GE Healthcare (GE Healthcare Bio-Sciences AB, SWEDEN). The fractions used to test the inactivation effect were quantified with the Bradford method prior to dilution to the concentrations described in the bioactivity-guided fractionation section.

### Shotgun mass spectrometry analysis and gene identification

The protein sample was isolated on a Tricine SDS-PAGE gel, and prior to overnight digestion with mass spectrometry (MS)-grade trypsin (1 μg), DTT and iodoacetamide were sequentially added to concentrations of 10 mM and 55 mM, respectively. The peptide samples from the in-gel digestion were analysed on an AB Sciex Triple TOF 5600-plus mass spectrometer interfaced with an Eksigent Nano LC Ultra (AB Sciex, USA). The samples were chromatographed using a 60-min gradient of 2–35% (buffer 0.1% [v/v] formic acid, 2% [v/v] acetonitrile, buffer 0.1 [v/v] formic acid, 90% [v/v] acetonitrile) after direct injection onto a 15-cm PicoFrit emitter (New Objective) packed to 15 cm with Magic C_18_ AQ 3-μm 200-Å stationary phase. The MS 1 spectra were collected over the range of 350–1,500 m/z for 250 minutes. The 30 most intense precursors with charge states 2–5 were selected for fragmentation. Then, the MS 2 spectra were collected over the range of 50–2,000 m/z for 100 minutes. The precursor ions were excluded from reselection for 15 seconds.

The MS/MS data were analysed using ProteinPilot (version 4.0.8085) and matched with AB Sciex 5600 Plus. The Swiss-Prot *R. palustris* protein database was used for all searches. The data analysis parameters were set as follows: trypsin as the enzyme; identification as the sample type; iodoacetamide as the Cys alkylation; no special factors or biological modifications; and a thorough identification search. The tryptic cleavage specificity, precursor ion mass accuracy and fragmentation mass accuracy were built-in functions of the Protein Pilot software. The paragon method was adopted to perform the database matching for protein identification. All reported data were based on 95% confidence for protein identification as determined by Protein Pilot (unused ProtScore 1.3).

Genomic DNA was extracted from *R. palustris* JSC-3b, and a pair of gene-specific primers was designed to amplify the Rhp-PSP gene sequence deduced from the shotgun mass spectrometry analysis, Swiss-Prot protein database search and genomic sequence of *R. palustris* JSC-3b. The primer sequences were as follows: forward primer, 5′-ATGGTTGAGCAGAAGCTCGC-3′; and reverse primer, 5′-GGCGACCTCGAACAGC-3′. The PCR product was cloned into the *p*EASY^TM^-T5 Zero Cloning vector (TransGen Biotech, Beijing, China) and verified by DNA sequencing (Beijing Genomics Institution, Beijing, China).

### Multiple sequence alignment of the YER057c/YjgF/UK114 family

The sequences of homologues of Rhp-PSP, i.e., PSPTO-PSP from *P. syringe*, YabJ from *B. subtilis*, TdcF from *E. coli*, Hp14.5 from humans, L-PSP from rats, UK114 from goats, and Hmf1 from *S. serevisiae* were obtained in UniProt with their UniProt No: Q88BE5, A7Z0H1, D9Z529, P52758, P52759, P80601, P40037. Their crystal structures were obtained in protein data bank RCSB PDB. The alignment was accomplished by ClustalW2 and colored with ESPpript3.0. The secondary structure of Rhp-PSP was predicted in JPred 4. The sequence alignment, the secondary structures, the highlights and the labels were integrated by Adobe Illustration 10.

### Rhp-PSP anti-TMV assays

To investigate the protective and curative effects of Rhp-PSP against TMV, Rhp-PSP was diluted with buffer D at concentrations of 50 and 100 μgmL^−1^, and the same concentrations of Ningnanmycin^39^ (Deqiang Biology Co., Ltd. (Harbin, China)) and BSA were set as check chemical and negative controls, respectively, for the half-leaf method. The protective and curative effects were calculated with the same formula used for the inactivation effect and described in the bioactivity-guided fractionation section.

Protective effect test of Rhp-PSP to TMV: 20 μL of Rhp-PSP, Ningnanmycin and BSA solutions were smeared on the right hand sides of the *N. glutinosa* leaves, and buffer D served as a control that was applied to the left hand sides of the same leaves. After 12 hours, the leaves were inoculated with 15 μL TMV solution (1 μgmL^−1^). Local lesion numbers were recorded 3 days after inoculation, and the formula was applied to the data to calculate the protective effects.

Curative effect test of Rhp-PSP to TMV: 15 μL TMV solution (1 μgmL^−1^) was inoculated onto the whole leaves of *N. glutinosa*, which were then washed with water and air-dried. Six hours after inoculation, 20 μL of Rhp-PSP, Ningnanmycin and BSA solutions were smeared on the right hand sides, and buffer D on the left hand sides of the inoculated leaves. Local lesion numbers were recorded for 3 days after inoculation, and the formula was applied to the data to calculate the curative effects.

### Statistical analysis

All of the data collected in this research were from four experimental repeats. The data are presented as the means ± the standard deviations, and significant differences between the treatments and the controls were determined with analyses of variance using SPSS Statistics 17.0.

## Additional Information

**How to cite this article**: Su, P. *et al.* Isolation of Rhp-PSP, a member of YER057c/YjgF/UK114 protein family with antiviral properties, from the photosynthetic bacterium *Rhodopseudomonas palustris* strain JSC-3b. *Sci. Rep.* **5**, 16121; doi: 10.1038/srep16121 (2015).

## Supplementary Material

Supplementary Information

## Figures and Tables

**Table 1:** Anti-TMV activities of Rhp-PSP and Ningnanmycin.

Compounds	Concentration (μg · mL^−1^)	Protective effect (%)	Curative effect (%)
Rhp-PSP	100	76.5 ± 2.9 a	78.3 ± 4.4 a
	50	65.8 ± 6.7 b	59.3 ± 4.1 b
Ningnanmycin	100	61.0 ± 3.2 b	54.5 ± 4.0 b
	50	45.0 ± 5.0 c	40.5 ± 5.4 c
BSA	100	4.5 ± 3.7 d	4.0 ± 2.6 d
	50	2.6 ± 2.2 d	2.8 ± 2.2 d

All results are expressed as the means ± the SDs, and n = 4 for all groups. The mean values followed by the same letters within each column were not significantly different according to Duncan’s multiple range tests (*P* < 0.05).
